# The Effect of the MEMS Measurement Platform Design on the Seebeck Coefficient Measurement of a Single Nanowire

**DOI:** 10.3390/nano8040219

**Published:** 2018-04-05

**Authors:** S. Hoda Moosavi, Danny Kojda, Maximilian Kockert, Saskia F. Fischer, Michael Kroener, Peter Woias

**Affiliations:** 1 Laboratory for Design of Microsystems, IMTEK, 79110 Freiburg, Germany; Michael.Kroener@gmx.com (M.K.); woias@imtek.uni-freiburg.de (P.W.); 2 Novel Materials Group, Physics Department, Humboldt University, 10099 Berlin, Germany; danny.kojda@helmholtz-berlin.de (D.K.); kockert@physik.hu-berlin.de (M.K.); sfischer@physik.hu-berlin.de (S.F.F.)

**Keywords:** thermoelectricity, Seebeck coefficient measurement, MEMS platform, nanowire characterization

## Abstract

In order to study the thermoelectric properties of individual nanowires, a thermoelectric nanowire characterization platform (TNCP) has been previously developed and used in our chair. Here, we report on a redesigned platform aiming to optimize performance, mechanical stability and usability. We compare both platforms for electrical conductivity and the Seebeck coefficient for an individual Ag nanowire of the previously-used batch and for comparable measurement conditions. By this, the measurement performance of both designs can be investigated. As a result, whereas the electrical conductivity is comparable, the Seebeck coefficient shows a 50% deviation with respect to the previous studies. We discuss the possible effects of the platform design on the thermoelectric measurements. One reason for the deviation of the Seebeck coefficient is the design of the platform leading to temperature gradients along the bond pads. We further analyze the effect of bonding materials Au and Pt, as well as the effect of temperature distributions along the bond pads used for the thermovoltage acquisition. Another major reason for the variation of the measurement results is the non-homogeneous temperature distribution along the thermometer. We conclude that for the measurement of small Seebeck coefficients, an isothermal positioning of voltage-probing bond pads, as well as a constant temperature profile at the measurement zone are essential.

## 1. Introduction

The Hicks and Dresselhaus prediction concerning the tremendous thermoelectric potential of nanowires [1] has redirected and refocused the field of thermoelectrics to nanotechnology. As a proof of concept, different research works have been implemented to assess the thermoelectric properties of nanowires. To gain insight into the correlation of the various physical effects and their influence on the thermoelectric performance, it is important to perform measurements on a single individual nanowire. This is enabled by making use of our previously-reported platform [2].

There are relevant platforms developed by different research groups, which make thermoelectric measurement of individual nanowires possible [3,4,5,6,7]. The main and unique feature of our platform is its TEM compatibility, which restricts us to small chip dimensions (<3 mm in the diagonal width of the chip). However, this constraint is the source of other issues, which are discussed in this work.

Thermoelectric measurements are very sensitive to the measurement conditions and, as a result, prone to error. Searching the literature, one would learn that the same material’s reported figures of merit are different, and even sometimes not reproducible from lab to lab. One reason for this might be the usage of different non-standardized measurement platforms. As an example of this challenge, we can recall the publications that reported on silicon nanowire’s figure of merit in the range of 0.6 to 1 [8,9]. Since then, although quite a large effort has been made on silicon nanostructures, such a result has not been reproduced [10,11,12].

As *ZT* = $\frac{\sigma S^{2}}{\kappa }T$, its measurement includes the experimental acquisition of three different temperature-dependent parameters: electrical conductivity (σ), thermal conductivity (κ) and Seebeck coefficient (*S*). The errors involved for each parameter step contribute to the final *ZT* value, but the Seebeck coefficient (thermoelectric power) is the most crucial parameter, as its contribution is quadratic.

There are publications available dealing with different possible sources of error in Seebeck coefficient measurement of different bulk and thin film materials [13,14]. Such systematic errors in a general measurement setup depend on the setup geometry and the thermocouple’s contact resistance. In this manuscript, we specifically report on Seebeck measurements on individual nanowires and possible sources of error.

We compare the Seebeck coefficient of three silver nanowires taken from the same batch, but measured the values by using two different platforms, called TNCP *a* and *b*. TNCP *a*, as has been addressed in [2,15,16,17]. TNCP *b* is the next generation of these characterization platforms, redesigned to enhance performance, mechanical stability and usability.

### 1.1. Nanowire Characterization Platforms: Platforms a and b

**Figure 1:**
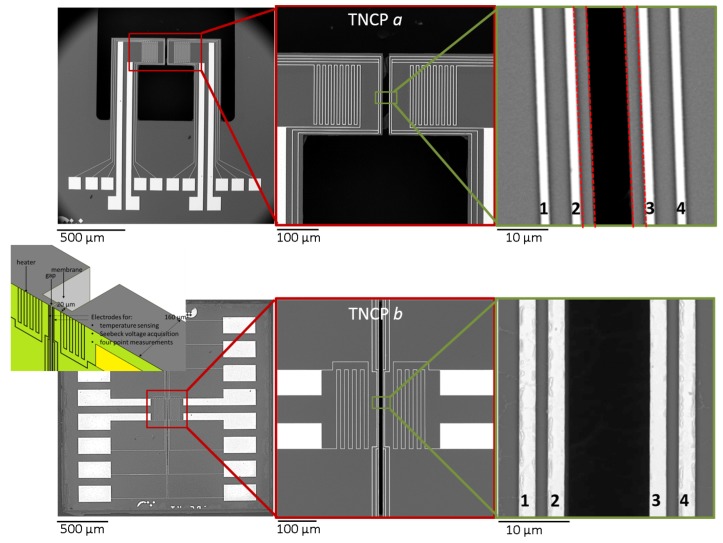
Two measurement platforms. Top: TNCP *a*, cantilever-based design; bottom: TNCP *b*, membrane-based design. The schematic inset illustrates Platform *b*’s cross-section to show its layout. In both cases, the electrodes are made of Pt.

Both thermoelectric nanowire characterization platforms, shown in Figure 1, are designed to enable a full thermoelectric characterization of a single nanowire. Furthermore, their transmission electron microscope (TEM) compatibility allows for a subsequent nanoscopic morphology, structural and chemical analysis of the same sample. The two platform designs follow two completely different strategies. Platform *a* (chip size: 2 mm × 2.6 mm × 200 μm) consists of two free standing cantilevers to assure proper thermal insulation of both sides of the attached nanowire. The cantilever’s length, width and thickness are 600μm, 200μm and 20μm, respectively. The cantilever tips are equipped with meander structures and measurement electrodes to be used as microheaters and temperature sensors, respectively. As freestanding cantilevers are very fragile and the bond pads have a small size and inter-distance, the use of TNCP *a* is challenging.

In the second phase of the project, the focus has been set on improving the design and fabrication method. As our TNCPs are intended to be used in collaborative projects, their robustness for shipping and handling is a significant factor. Therefore, in the new design, the free standing cantilevers are substituted by membranes to increase the mechanical stability of the platform.

TNCP *b* comprises a long front side gap (1000 μm) in a suspended membrane, dividing the platform into two halves to provide the thermal insulation of the suspended nanowire at both sides. The gap widths realized vary between 8,10 and 12 μm, allowing for investigation of nanowires with different lengths. Right below the front side gap, a wide back side gap (with lateral dimensions of 100 μm × 1000 μm) is implemented, to generate a stack layer/Si membrane structure, with a thickness of 300 nm/20 μm, respectively [18]. This increases the field of view during the tilted mode TEM investigation. As its ancestor, TNCP *b* is equipped with four-point measurement electrodes, temperature sensors and microheaters, on both sides of the gap.

**Figure 2:**
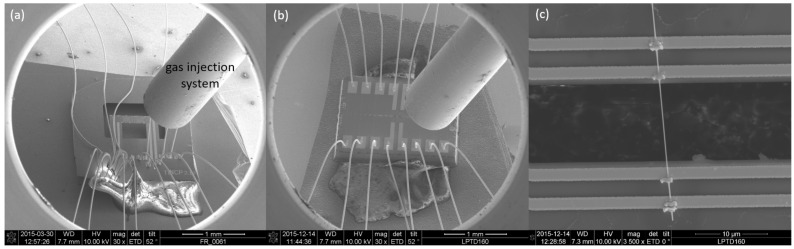
(**a**,**b**) TNCPs *a* and *b* in the focused electron beam-induced deposition (FEBID) chamber. The injection system introduces the precursor gas to the chamber. Symmetric bond pads provide a platform for easier wire bonding, and also, FEBID is possible without disturbing wires over the nanowire of interest. (**c**) The contacted Ag nanowires on Platform *b*.

Another room for the design improvement relies on the bond pads; the small bond pads, all being accommodated at one side of the chip, made the further wire bonding process challenging in Platform *a*. The symmetric, large bond pads are therefore another distinguishing factor of Platform *b* (see Figure 1 and Figure 2).

The chip size is reduced to 2 mm × 2 mm × 160 μm, to make the platform even more compatible with conventional TEM holders. The design details and fabrication technique, based on the utilization of 110 silicon wafers, are discussed elsewhere [19,20,21,22].

### 1.2. Technical Differences in Design between Platforms a and b

The thermovoltage is collected over the inner electrodes (Electrodes 2 and 3) in both platforms (Figure 1), as this is the region being analyzed in TEM. In Platform *a*, Electrodes 1 and 4 are used as temperature sensors. In Platform *b*, however, the temperature sensors are implemented in the same electrode where the thermovoltage is measured (Electrodes 2 and 3), in order to enhance the accuracy of the Seebeck coefficient measurement.

Another technical difference between the two platforms is the region of TEM compatibility. The very narrow silicon rim between the inner measurement electrode and cantilever tip of Platform *a* (confined in dotted lines in Figure 1) limits the field of view of the suspended nanowire in TEM. Additionally if the deposited nanowire happens to touch this part, the interpretation of the measured data becomes difficult, as this contact might have an impact on the measurement, although an electrically isolating layer is realized on top of the silicon substrate. In Platform *b*, this rim is removed, and at the same time, extreme care is taken to avoid any underetching of the electrodes. The fabrication challenges are discussed in [18].

## 2. Results

To obtain the thermovoltage, microheaters are used to generate a temperature gradient along the nanowire attached to Platform *b*. The temperature gradient and the subsequent resulting thermovoltage are detected at the inner electrodes.

The measured Seebeck coefficient (the ratio of the thermovoltage to the temperature gradient) is reported with respect to Pt (as the electrode): $S_{Ag,Pt}=S_{Ag}-S_{Pt}=\frac{-\delta V_{S}}{\delta T}$.

To avoid systematic errors in the Seebeck coefficient acquisition, measurements on Platform *b* are performed in the same cryostat device (KONTI-IT) and at the same pressure (helium atmosphere at 1 bar) as the former measurements on Platform *a*.

**Figure 3:**
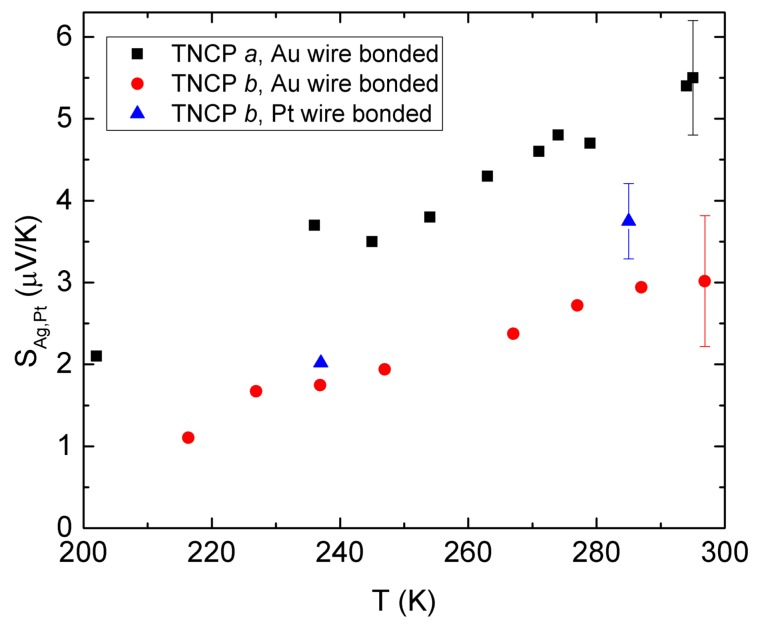
Ag nanowire Seebeck coefficient (with respect to Pt electrodes) and its temperature dependency, measured by TNCP *a* (data adapted from [16]) and TNCP *b*. The wires that bond the TNCP *b* pads to the peripheral electrodes are made from Pt or Au. The maximum error bar is depicted and was determined by fitting the minimum and maximum slopes, as a worst-case result (refer to Figure 8). Other methods (square root of covariance matrix) may lead to considerably lower errors.

Figure 3 shows the measured Seebeck coefficient using both platforms. The results of the Ag nanowire Seebeck coefficient (with respect to Pt) measured by TNCP *b* however indicate an (approximately) 50% deviation from the data taken by TNCP *a*. As an example, at T=297 K, our measured *S* is 3.02 ± 0.8 μV/K, while it has been reported to be 5.5 ± 0.7 μV/K making use of TNCP *a* at the ambient temperature of 295 K [16]. As shown by the blue points, changing the material of the bond wire from Au to Pt in TNCP *b*, 22% higher *S* is measured at 285 K. The bulk value is 6.79 μV/K for room temperature, as reported in [23].

This difference in the obtained data, using both platforms, can be interpreted taking two possibilities into account: emergence of additional, unintended thermovoltages and/or erroneous temperature sensing.

## 3. Discussion

**Figure 4:**
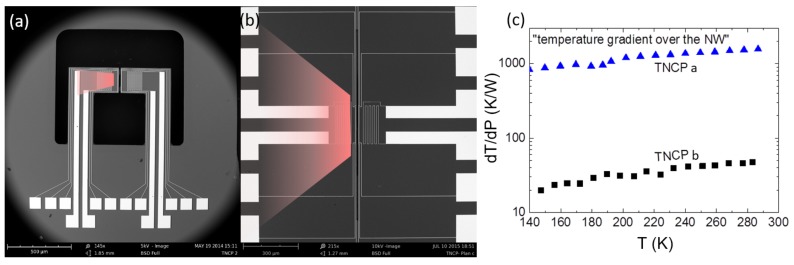
(**a**,**b**) The generated heat distribution in both platforms. Due to the design, heat is more concentrated in Platform *a*. (**c**) Temperature gradient across the nanowire site in both platforms.

To seek the difference in the Seebeck value measured in TNCP *b*, we reconsider both platform designs. Platform *a*, as all the heat generated by the heater is concentrated in the cantilevers, benefits from a localized heat area. Additionally, the bond pads are all far from the heating zone, placed at the same thermal level. Platform *b* lacks such a possibility, and the generated heat can penetrate into the heated halve of the chip (see Figure 4a,b). This lack of heat concentration is clearly shown in the diagram depicted in Figure 4c. To achieve similar temperature gradients across the gaps, one magnitude of order more power must be applied to the heater in the new TNCP.

**Figure 5:**
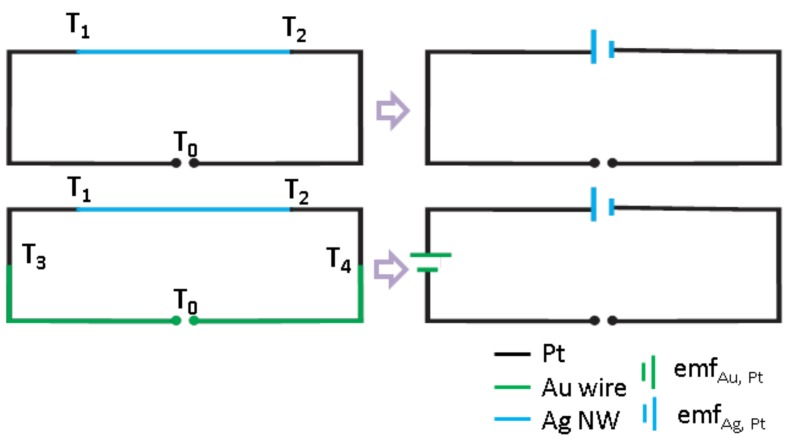
Different possible Seebeck interfaces in a gold wire-bonded TNCP. The thermally-induced electromotive force (emf) can occur in two metal joints in the case of a temperature gradient.

Now considering this fact, it may happen that the generated heat in TNCP *b* distributes through the whole area at the heated side and reaches even the bond pads at the chip edge. The Pt bond pads are bonded with gold wires. As a consequence of the temperature gradient at the joint of two different metals, an additional thermovoltage is generated at the interface, which contributes to the nanowire’s measured Seebeck voltage. This possibility is schematically illustrated in Figure 5. $T_{1}$ and $T_{2}$ are the temperature values at both sides of the nanowire. $T_{3}$ and $T_{4}$ represent the temperature at the bond pads.

If that is the case, the temperature gradient at the bond pad can be calculated, considering the absolute thermoelectric powers of $S_{Pt}=-5.28\;\mu$V/K and $S_{Au}=+1.94\;\mu$V/K [23]. In our measurement the temperature sensors indicate the gradient across the nanowire to be δT=4K. Assuming that the $S_{Ag,Pt}=5.5\;\mu$V/K measured with TNCP *a* is correct, we had to collect break $V_{s}=-22\;\mu$V in TNCP *b*. This, however, is measured to be $V_{s}=-10.5\;\mu$V. The missing thermovoltage of $V_{s,missing}=-11.5\;\mu$V would correspond to a temperature gradient of 1.6 K between the bond pads.

To investigate the probability of such a heat distribution over the bond pads, the heat performance of TNCP *b* is simulated using a COMSOL 3D FEM coupled thermal and electrical module. Considering the real measurement condition in which He is used as the carrier gas, the simulation was performed taking the chip enclosed by a helium (He) box of dimensions 2010×2010×290μm${}^{3}$, at a pressure of 1 bar.

To get a temperature gradient of 4 K across the gap, a heater power of 68 mW is required in the experiment. To generate the same δT, the heater power in the simulation is 136.28 mW. The reason that the experiment and simulation values do not match is due to the simplifications applied during the simulation. This will be addressed in more detail in the following.

**Figure 6:**
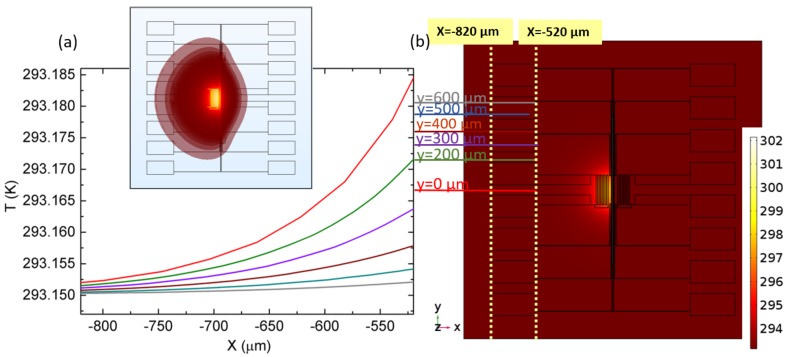
FEM simulation on Platform *b*; electrical energy dissipation in the heater structure causes the subsequent heat increase at one side of the gap. (**a**) The heat distribution across the bond pad. The inset shows the isothermal contour of the temperature distribution over the platform. (**b**) xy view of the simulated chip. Getting farther from the heater, the bond pads are more intact (compare the red and grey lines in both diagrams in (a) and (b)).

Figure 6 shows the line scan of the heat contribution at different cross-sections of the simulated chip, across the bond pads.

As shown in Figure 6, there is a temperature gradient over the bond pads, at the heated side of the gap, compared to the other side, where the bond pads’ temperature is equal to the heat sink temperature (i.e., 293.15 K). This is more vivid in the inset isothermal contour. However, this temperature gradient is much smaller than our calculated suspicious value (δT=1.6 K).

To calculate the additional thermovoltage contribution, using this simulated set of data, we consider the temperature gradient across the line y=0. In the experiment, this bond pad is the one for driving the heater, and no thermovoltage is collected here; however, as it is the worst possible case, to be on the safe side, this one is considered. Based on the simulation data, there is a temperature gradient of 0.035 K over this scan line, on the surface of the TNCP.

By taking $T_{4}$ the temperature at the heat sink (and the bond pad temperature at the cold halve of the chip), $T_{3}$ the maximum temperature at the bond pad (in the heated halve of the platform) and $T_{1}$ and $T_{2}$ the temperatures at the hot and cold side of the nanowire, respectively, we will have:

(1) $$V_{S}=-\left(\left(S_{Au}-S_{pt}\right)\left(T_{3}-T_{4}\right)+\left(S_{Ag}-S_{pt}\right)\left(T_{1}-T_{2}\right)\right)$$

Therefore, including the simulation value, the measured thermoelectric voltage contains an additional 0.25μV component [$V_{S}=-(0.25\;\mu V+S_{Ag,Pt}\delta T_{1,2})$]. This value is much less than the missing one −11.5μV.

The reason that the simulation does not represent the experimental system perfectly could be due to the simplifications applied, compared to the actual case. The TNCP is glued to the top of a silicon piece using a silver conducting paste. The silicon piece itself is mounted on a ceramic sample holder. For the sake of simplicity, these are not included in the simulation.

Considering this, we regarded the simulation as an indication for the presence of some heat distribution over the bond pads, although it is less than the actual value.

To prevent the unfavorable effect of the bond pads’ gradient temperature distribution on measurements, three remedies are conceivable:

- Placing the bond pads responsible for Seebeck voltage collection both at the same temperature level, i.e., at the same side of the platform (like Platform *a*), ensures their uniform temperature coverage. This solution is however not a good one, as in that case, the ease of wire bonding is compromised.
- An improvement can be achieved by performing wire bonding with platinum wires. As the Seebeck coefficient of bulk platinum and its thin film are “almost” identical [24], the additional Seebeck voltage at their interface is omitted.
- If the platform’s metal structures are made of gold, then the conventional gold wire bonding can be used. Implementation of gold wire bonding is technically easier than platinum. Still, it must be taken into consideration that in this case, also there might be some deviations for the Seebeck coefficient of the thin film and wires. Furthermore, the Seebeck measurement reference will be gold instead of platinum.

As mentioned earlier, the temperature sensor electrodes and the electrodes at which the Seebeck voltage is collected are different in TNCP *a*. This can be considered as another source of error. Based on the helium atmosphere simulation data (performed on TNCP *b*), the sum of the temperature differences in the sensor and measurement electrode, at both the hot and cold side, is equal to 0.046 K. Again, to remind, this value is simulated using a simplified situation compared to the actual case. This error is avoided in the TNCP *b* design.

**Figure 7:**
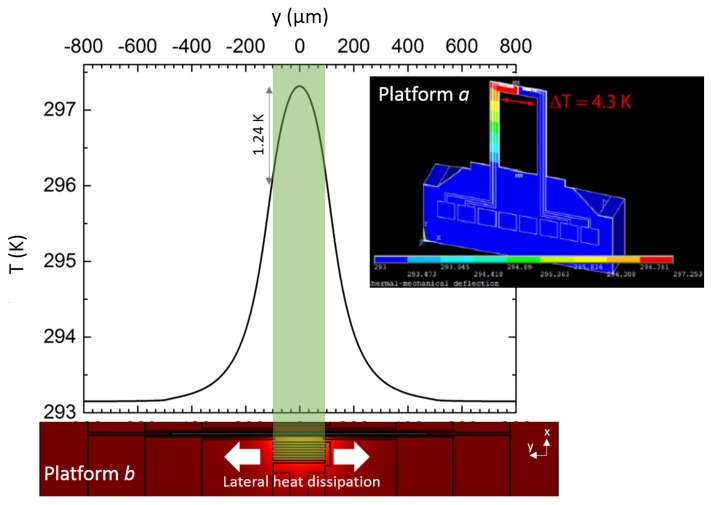
Temperature distribution along the thermometer for the hot side of TNCP *b*. The inset shows the simulation, made on an older generation of Platform *a*, with different dimensions, compared to the one that is implied in this work. However, as the basic design is cantilever-based, this comparison is valid. The inset shows data from [25], with permission from Elsevier.

One other critical point to think of is the temperature distribution along the thermometers. Due to geometrical boundary conditions, it is more homogeneous in TNCP *a* than in TNCP *b*, which is evidenced by the simulation shown in the inset of Figure 7. The graph in Figure 7 shows the temperature line scan along the thermometer (y-direction) at the hot side of Figure 6’s simulation. Due to the lateral heat dissipation in TNCP *b*, there is no homogeneous temperature profile in the measurement zone (the light green contour). Thus, for centered nanowires, the temperature is underestimated and for wires close to the edge of the measurement zone overestimated. Integration of the Gaussian fit of the temperature distribution along y=−100 to y=100μm (the measurement area) reveals that the mean temperature, sensed by the thermometer, is equal to $T_{mean}=296.8815$ K. As the nanowire can be placed at any position on the thermometer, the maximum error in temperature sensing at the hot side is in the order of approximately 0.43 K, i.e., ($T_{max}-T_{mean}$). If we have a 0.43 K deviation of an approximately 4 K temperature difference along the gap, this gives an error of about 11% in the overall nanowire temperature sensing. This concludes that the accuracy of the temperature in TNCP *a* is higher.

### 3.1. Improving the Seebeck Measurement Using TNCP b

To cancel out the effect of the additional thermovoltages emerging in the results, we have bonded TNCP *b* by making use of Pt bond wires. The results, depicted in Figure 3 and Table 1, indicate an improvement in the Seebeck coefficient determined by TNCP *b*, while Pt wires are utilized, but still, the same data as with TNCP *a* are not realized. This can be explained as follows: There can be a small difference between the absolute Seebeck coefficient of the bond wire and of the thin platinum bond pad around room temperature, as reported in [24]. This difference gets larger with decreasing bath temperature. Therefore, a small temperature gradient along the system bond pad (Pt)-bond wire (Pt) can lead to an additional thermovoltage, which results in higher offsets. This offset is even higher with the system bond pad (Pt)-bond wire (Au).

It is important to mention that even the type of the bond wire can enhance the measurement accuracy; still, one source of error is unsolved in TNCP *b*. In TNCP *b*, the measured thermovoltage offset value was measured to be higher than the nanowire thermovoltage, as shown in Table 1.

This offset can essentially be problematic, while determining the thermovoltage of the nanowires with a low Seebeck coefficient (metallic nanowires).

The main reason lies in the placement of the contacts, which are not at the same temperature level. This can be reduced to less than 10μV as demonstrated by TNCP *a* in which the contacts were placed at the same temperature level.

### 3.2. Seebeck Measurement: A General Note on Errors and Uncertainties

**Figure 8:**
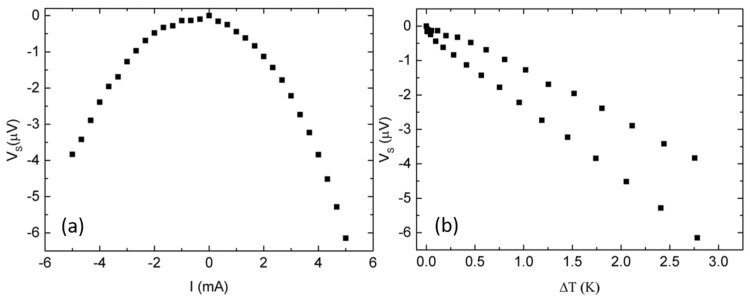
Thermovoltage at an ambient temperature of 237 K, as a function of (**a**) heater current and (**b**) temperature difference. The measurement data shown are corrected by a thermovoltage offset of −59μV, which is typical in TNCP *b* because the contacts are not placed at the same temperature level.

In the following, we present four general errors that can arise during the nanowire thermovoltage acquisition procedure:

- During the fabrication process of both platforms, as presented in [2,21], a 20 nm-thick Ti layer is sputtered as adhesion promoter for the Pt thin film. Therefore, the Seebeck coefficient is in fact measured with respect to these two metal layers as a reference. However, as the Seebeck coefficients of Ti and Pt are comparable, they cancel each other out [26]. Therefore, this is not a source of error in our case. In the case of utilizing other adhesion promoters, or other metals as electrodes, this key point must be taken into account.
- Good electrical and thermal connection is a prerequisite in the Seebeck measurements. Our technique is focused electron beam-induced deposition (FEBID); however, there is an uncertainty about the inevitable involvement of unwanted elements (such as carbon) deposited during the contacting process, which may be different from each deposition in some percentages. This contributes to some measurement errors.
  In this work, we estimated this error to be very low, justified by our research on thermal resistances of FEBID contacts, which is much smaller than that of the Ag nanowires, as published in [16]. Therefore, we assume the nano-sized Pt FEBID contacts and the Ag nanowire to experience the same temperature so that no additional thermovoltages are taken into account.
- The contact resistance between the heater pads and the wires is probable due to the intermetallic compound growth during wire bonding [27]. Considering this, when electrical current is applied to the heaters, they will also heat up and change the local temperature. To avoid this source of error, it is a good idea to design the heaters’ bond pads far from the bonds at which thermovoltage is collected.
- In our measurements, to obtain the thermovoltage, the heater is swept from −5 mA to +5 mA. Figure 8 shows that the thermovoltage collected for the same heater powers in TNCP *b*, corresponding to the two current polarities, does not fit completely. This is a source of error indicating that the wait time before measurement has not been adequate. This problem is dominant in TNCP *b*, due to its non-concentrated thermal performance. This can be reduced by considering an appropriate wait time after assigning the heater current. The drawback is the increase of measurement time. This has been no issue for TNCP *a*, as shown in [16]. For that case, the equilibrium after the heater power adjustment is reached almost instantly.

## 4. Materials and Methods

The silver nanowires used are synthesized by reducing silver nitrate ($AgNO_{3}$) with ethylene glycol (EG). The details of the synthesis are reported elsewhere [16]. The TNCP is glued to a 16-pin ceramic sample holder. Then, it is wire bonded using 25μm gold wires. To assemble the nanowire, a droplet of the aqueous medium containing the suspended nanowires is drop-cast on a photoresist-grooved substrate. After the droplet dries, the nanowires can be picked by a micromanipulator arm and placed on the measurement site on the TNCP, under a high resolution optical microscope.

The focused electron beam-induced deposition (FEBID) technique is used as an electrical contact enhancement tool [28]. To this aim, a platinum-rich precursor gas is injected into the chamber, using the gas injection system. As a result, the nonvolatile products deposit at the focused electron beam area, while the volatile fragments are exhausted.

**Figure 9:**
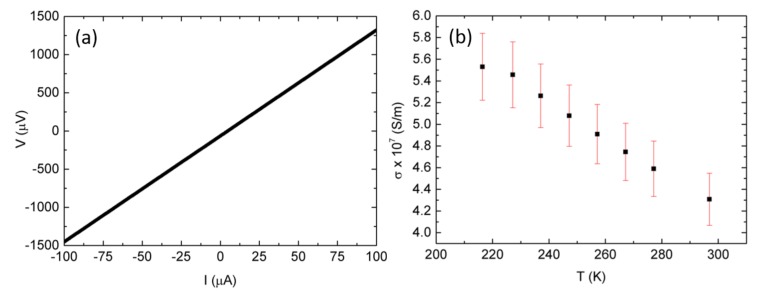
Ag nanowire electrical characterization using TNCP *b*. (**a**) I-V curve of the Ag nanowire at *T* = 297 K. The linear trend shows an ohmic contact. (**b**) Ag nanowire electrical conductivity. The conductivity decrease versus the temperature increase is a typical behavior of metals.

After nanowire assembly and contacting, the chip holder is loaded into the cryostat setup (KONTIIT). The I-V curve, measured by means of four point configuration, shows a linear trend, as shown in Figure 9a, which is proof of an appropriate electrical contact. The resistance data of the nanowire at different temperatures is used to assess its electrical conductivity, as depicted in Figure 9b. The electrical characterization of the nanowire in TNCP *a* is reported in [16].

The nanowire assembled with TNCP *b*, with dimensions $l_{NW}=14.37\pm 0.78\;\mu$m and $d_{NW}=175\pm 1$ nm, shows an electrical conductivity of $\left(4.31\pm 0.24\right)\times 10^{7}$ S/m at *T* = 297 K. The electrical conductivity of the silver nanowire, with dimensions $l_{NW}=14\pm 1\;\mu$m and $d_{NW}=150\pm 3$ nm, measured using TNCP *a*, is reported to be equal to $\left(4.0\pm 0.2\right)\times 10^{7}$ S/m at room temperature [16]. The measured σ values in both platforms correspond well. It is important to indicate that the dimensions of the nanowire assembled with Platform *b* are achieved by means of the SEM image. The other nanowire, attached to TNCP *a*, was studied in TEM, and its dimensions have been achieved by means of image processing of the TEM data.

**Figure 10:**
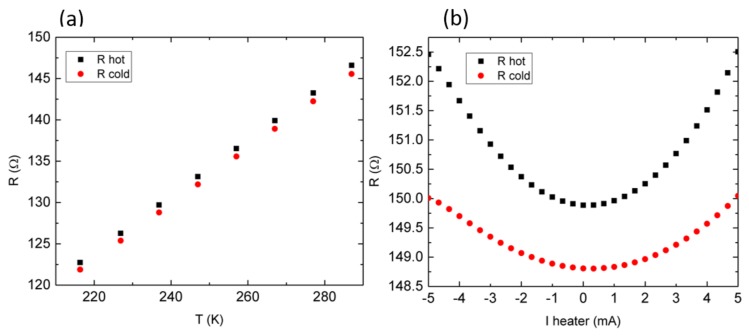
(**a**) Thermometers’ “hot” and “cold” electrodes’ calibration. (**b**) Thermometers’ resistance evolution with respect to the current applied to the microheater, at room temperature.

To measure the Seebeck coefficient, the temperature sensors must be calibrated. To do so, after chamber temperature stabilization, the resistance of both thermometers with respect to the bath temperature is recorded, as shown in Figure 10a. Knowing the slope value of this diagram, any further change in the thermometer electrodes’ resistance (Figure 10b) can be assigned to a related temperature change.

## 5. Error Propagation Considerations and Final Conclusions

In this study, the Seebeck coefficient of three Ag nanowires, from the same synthesis batch, measured by means of two different characterization platforms, are compared. The nanowire assembled with TNCP *b* is contacted by the FEBID technique, and the one on TNCP *a* is laid on top of the Pt electrode, without FEBID contacting. This introduces an error of $\Delta \delta V_{a,b}$ to the results of both TNCPs, due to the uncertainties in the contacting procedure. As the contact size is small, the thermovoltage uncertainty at these areas is also small and can be neglected. However, to be exact, we consider them in this discussion. The suffixes “*a*” and “*b*” correspond for TNCPs *a* and *b*, respectively.

As simulation shows, some temperature distribution arises for the bond pads in TNCP *b*. This adds an error $\Delta \delta V_{b}$ to the measured thermovoltage, which is equal to 0.25μV for a δT=4 K across the nanowire. Also for the same δT, a temperature sensing error of $\Delta \delta T_{b}=0.43$ K is deduced from the simulation data at the hot thermometer side. On the other hand, as temperature sensing takes place at the adjacent Seebeck electrode, a temperature measurement error may be introduced to the results of the TNCP *a*; $\Delta \delta T_{a}$, which is in the range of 0.046 K for δT = 4 K across the nanowire (based on the simulations performed on Platform *b*).

Based on common error propagation analysis, one would derive ΔS by taking the partial deviation of the independent parameters ΔV and ΔT as follows:

(2) $$S=-\frac{\delta V}{\delta T}\Rightarrow \Delta S=\sqrt{{\left(\frac{\partial S}{\partial \delta V}\right)}^{2}{\left(\Delta \delta V\right)}^{2}+{\left(\frac{\partial S}{\partial \delta T}\right)}^{2}{\left(\Delta \delta T\right)}^{2}}$$

(3) $$\Delta S_{TNCP_{a}}=\sqrt{{\left(\frac{1}{\delta T}\right)}^{2}{\left(\Delta \delta V_{0}+\Delta \delta V_{a,b}\right)}^{2}+{\left(\frac{-\delta V}{\delta T^{2}}\right)}^{2}{\left(\Delta \delta T_{0}+\Delta \delta T_{a}\right)}^{2}}$$

(4) $$\Delta S_{TNCP_{b}}=\sqrt{{\left(\frac{1}{\delta T}\right)}^{2}{\left(\Delta \delta V_{0}+\Delta \delta V_{a,b}+\Delta \delta V_{b}\right)}^{2}+{\left(\frac{-\delta V}{\delta T^{2}}\right)}^{2}{\left(\Delta \delta T_{0}+\Delta \delta T_{b}\right)}^{2}}$$

$\Delta \delta V_{0}$ and $\Delta \delta T_{0}$ are the common errors. $\Delta \delta V_{0}$ is due to the device (Keithley 2182A) collecting the voltage. To reduce the measurement error at each bath temperature, the thermovoltage is measured five times, and the standard deviation is reported as this common error. $\Delta \delta T_{0}$ emerges due to the sensor calibration and also depends on the internal setup thermometer.

The common errors have been considered in the reported Seebeck coefficient values, as the error of the linear fit of thermovoltage with respect to the temperature. By temporarily excluding the common errors, the effective errors (which stem from the chip design) in both results can be derived as follows:

(5) $$|\Delta S_{TNCP_{a}}{|}_{eff}=\left(\frac{1}{\delta T}\right)\sqrt{{\left(\frac{\delta V}{\delta T}\right)}^{2}{\left(\Delta \delta T_{a}\right)}^{2}}$$

(6) $$|\Delta S_{TNCP_{b}}{|}_{eff}=\left(\frac{1}{\delta T}\right)\sqrt{{\left(\Delta \delta V_{b}\right)}^{2}}+\left(\frac{1}{\delta T}\right)\sqrt{{\left(\frac{\delta V}{\delta T}\right)}^{2}{\left(\Delta \delta T_{b}\right)}^{2}}$$

As is shown in Equations (5) and (6), the error from the temperature measurement is dominant as it is multiplied by the Seebeck factor.

To get an idea about the relative magnitude of $|\Delta S_{TNCP_{a}}{|}_{eff}$ and $|\Delta S_{TNCP_{b}}{|}_{eff}$, the FEM simulation data are used. We can compute the contribution of erroneous temperature sensing to the measured Seebeck coefficient, using TNCP *a*, by considering δT=4 K, ΔδT=0.046 K. This gives $|\Delta S_{TNCP_{a}}{|}_{eff}=0.07\;\mu$V/K, using Equation (5), while considering the experimentally-achieved $\frac{\delta V}{\delta T}$, i.e., $S_{Ag,Pt}$. To calculate the value of erroneous thermovoltage at the bond pads, as well as the non-homogeneous temperature distribution along the thermometer in the TNCP *b* results, taking $\Delta \delta V_{b}=0.25\;\mu$V, δT=4 K and $\Delta \delta T_{b}=0.43$ K, gives $|\Delta S_{TNCP_{b}}{|}_{eff}=0.38\;\mu$V/K, using Equation (6).

To conclude, nanowire Seebeck coefficient measurement is very sensitive and requires extra care both for thermovoltage acquisition and temperature sensing. TNCP *b* is favorable for the handling, processing and contact preparation of individual nanowires because the platform is mechanically more stable and provides more space for bonds. It is very suitable for measurements of the electrical and thermal conductivity. Regarding Seebeck measurements, it can be well applied to nanowire materials of larger Seebeck coefficients. However, due to the design of the platform, thermovoltages of only a few μ V cannot be securely resolved. There are two main reasons for this:

- As the bond contacts are not placed at the same temperature level, an additional thermovoltage is measured at the position of the bond pad and the wires bonding the chip into the peripheral electrodes. Therefore, to measure small Seebeck coefficients, isothermal positioning of all voltage probing bond pads is essential.
- For proper temperature sensing, it is not only essential to measure the temperature at the electrode that collects the nanowire thermovoltage, but also, the temperature along the thermometer, across the measurement zone, must be homogeneous. The sensitivity for the temperature reading in a characterization platform is the most important key point, as any temperature uncertainty would be magnified by a factor of thermovoltage, as shown in Equations (5) and (6).

## Figures and Tables

**Table 1:** Ag nanowire Seebeck coefficient realized using different measurement platforms, wire bonded by Au and Pt wires.

Measurement Tool	T (K)	S (μV/K)	ΔS (μV/K)	*V*${}_{\mathrm{offset}}$ (μV)	$V_{S,\mathrm{Ag}}$ (μV)
TNCP *a*, Au wire bonded	279	4.7	0.7	<10	
TNCP *b*, Au wire bonded	287	2.9	0.8	−84	16
TNCP *b*, Pt wire bonded	285	3.8	0.46	58	14

## Abbreviations

The following abbreviations are used in this manuscript:

TEM	transmission electron microscope
TNCP	thermoelectric nanowire characterization platform
FEM	finite element method
NW	nanowire
emf	electromotive force
